# Epicardial Pulsed Field Ablation of Ganglionated Plexi: Computational and Pre-Clinical Evaluation of a Bipolar Sub-Xiphoid Catheter for the Treatment of Atrial Fibrillation

**DOI:** 10.3390/bioengineering11010018

**Published:** 2023-12-24

**Authors:** Barry O’Brien, John Reilly, Ken Coffey, Ana González-Suárez, Piotr Buchta, Piotr P. Buszman, Karolina Lukasik, Jason Tri, Martin van Zyl, Samuel Asirvatham

**Affiliations:** 1 AtriAN Medical Ltd., Unit 204, Business Innovation Centre, Upper Newcastle, H91 W60E Galway, Ireland; 2 Translational Medical Device Laboratory, School of Medicine, University of Galway, H91 YR71 Galway, Ireland; 3 Valencian International University, Valencia, Spain; 4 3rd Department of Cardiology, Faculty of Medical Sciences in Zabrze, Medical University of Silesia, 40-055 Katowice, Poland; 5 Silesian Center for Heart Diseases, 41-800 Zabrze, Poland; 6 Center for Cardiovascular Research and Development, American Heart of Poland, Kostkowice, Poland; 7 Andrzej Frycz Modrzewski Kraków University, 30-705 Kraków, Poland; 8 Mayo Clinic, Rochester, MN 55905, USA; 9 Royal Jubilee Hospital, University of British Columbia, Victoria, BC, Canada

**Keywords:** atrial fibrillation, ganglionated plexi, pulsed field ablation, epicardial, subxiphoid

## Abstract

Epicardial pulsed field ablation (PFA) of ganglionated plexi (GPs) is being explored as a potential treatment for atrial fibrillation. Initial work using open-chest access with a monopolar ablation device has been completed. This study describes the early development work for a device that can be used with subxiphoid access and deliver bipolar ablation pulses. Electric field computational models have been used for the initial guidance on pulse parameters. An in vivo assessment of these ablation parameters has been performed in an open-chest canine study, while subxiphoid access and navigation of the device has been demonstrated in a porcine model. Results from this acute study have demonstrated the promising potential of this approach.

## 1. Introduction

Pulmonary vein isolation (PVI), through catheter ablation, is the current interventional standard for treating atrial fibrillation (AF) in patients with drug-refractory paroxysmal and persistent atrial fibrillation [1,2]. Both radiofrequency (RF) heating and cryogenic balloon cooling have been widely used and demonstrated to be generally equivalent in terms of their efficacy outcomes [3]. Additionally, the more recently introduced laser balloon has provided outcomes generally equivalent to RF and cryoballoon ablation [4,5]. However, 1-year success rates have been only modest at 65–70%, and these rates continue to drop slowly over time; the FreezeAF study showed that for paroxysmal AF patients at a 30-month follow-up, the single procedure success rate was only 40% and 42% for RF and cryoballoon ablation, respectively [6]. This modest performance, combined with the serious risks around atrioesophageal fistula [7] and phrenic nerve injury [8], have been the main drivers behind the exploration and development of pulsed field ablation (PFA) as a nonthermal alternative.

**Figure 1:**
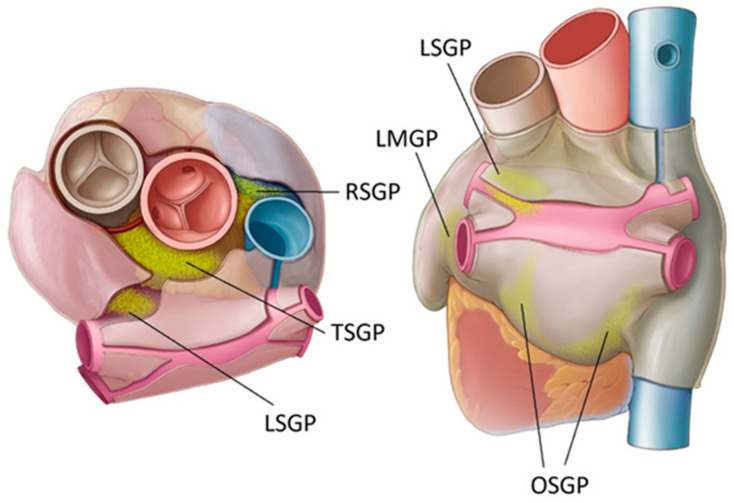
Location of key atrial ganglionated plexi (GPs) in humans. Oblique sinus (OS), right superior (RS), transverse sinus (TS), left superior (LS), and ligament of Marshall (LM).

Several PFA PVI clinical studies have now been completed, including the MANIFEST registry [9] and the ADVENT trial [10], which was a direct comparison of PFA with conventional thermal ablation. A recent meta-analysis has also compared periprocedural complications and AF recurrence rates for PFA with thermal approaches [11]. These studies are all demonstrating that PFA has an excellent safety profile, significantly reducing the concerns around esophageal and phrenic nerve injuries. However, efficacy outcomes are not significantly different compared to conventional methods; the 1-year success rate in the ADVENT trial was 71.3% for conventional thermal ablation and 73.3% for PFA. Given the very high rates of vein isolation reported acutely and at the 3-month follow-up in prior PFA trials [12,13], achieving higher rates of AF-free survival will likely require further improvements to PFA PVI or a strategy which goes beyond the pulmonary veins. In this context, the observations around autonomic modulation during PVI are interesting; evidence to date indicates that endocardial PFA PVI is not modifying or ablating the epicardial ganglionated plexi (GPs) [14,15], as so happens during thermal ablation. It has been widely recognized that the collateral thermal damage to some of the GPs may, in part, contribute to outcomes of endocardial PVI [16,17]. The absence of such damage with endocardial PFA may, in part, explain the ceiling in efficacy outcomes. A technique for epicardial PFA of GPs is currently in development [18]; the safety and feasibility of this approach has already been demonstrated preclinically [19] and clinically [20] in open-chest surgical settings. Figure 1 shows the GP sites targeted in these studies, using two purpose-designed catheters and a pulsed field generator (AtriAN Medical Ltd., Galway, Ireland). This current study presents the findings of electric field models and initial preclinical work to adapt this open-chest treatment for a minimally invasive subxiphoid approach. Additional objectives were to change the energy delivery from the monopolar configuration (used in the open-chest setting) to a bipolar arrangement, as well as consolidate the catheter designs into one configuration.

## 2. Materials and Methods

### 2.1. Ablation System Designs

**Figure 2:**
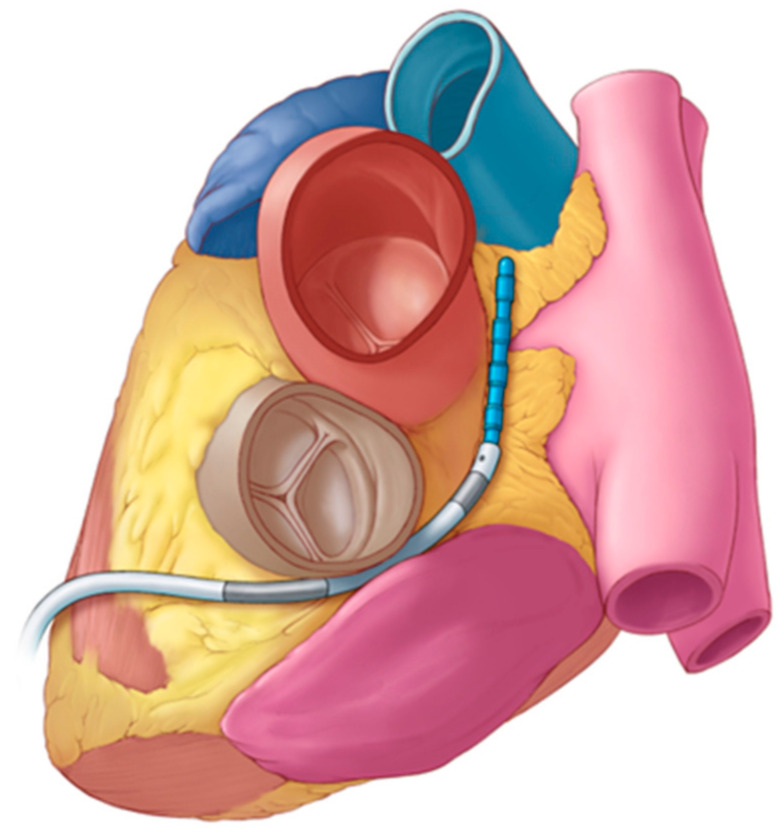
Illustration of the bipolar subxiphoid device (blue) deployed in the transverse sinus using a steerable access sheath to assist with navigation within the pericardial space, after obtaining anterior subxiphoid access.

The monopolar open-chest system includes the *Glove* catheter for treating GPs within the oblique sinus and the *Finger* catheter used for all other atrial GP locations. The *Glove* device has six irrigated electrodes arranged in two parallel rows of three electrodes each, while the *Finger* device has four irrigated electrodes in a conventional quadripolar configuration. The wider configuration of the *Glove* makes it possible to treat the GPs at both the inferior left and inferior right sides of the oblique sinus at once, rather than needing to position the device individually at these left and right GPs. All electrodes are insulated on the nontreatment surface to aid in the directional delivery of the electric field. In both devices, the electrodes are connected and operate together in a monopolar manner, with a dispersive return pad on the skin of the subject’s lumbar region. The single subxiphoid bipolar prototype device is a quadripolar configuration, again with irrigation through the electrodes and with electrical insulation on the nontreatment surfaces. Each electrode is individually wired, allowing for multiple bipolar arrangements between the four electrodes. This device has a lower profile than the open-chest tools and is designed for compatibility with commercially available 8.5 Fr steerable epicardial access sheaths. Figure 2 schematically illustrates the bipolar device navigated to the transverse sinus location.

The monopolar and monophasic pulse delivered is 1000 V in amplitude and a width of 100 µs. Saline (0.9%) is irrigated through the electrodes at a flow rate of 2 mL/min. The same parameters are used for the bipolar pulses, which are also delivered in a monophasic manner.

### 2.2. Electric Field Modeling

**Figure 3:**
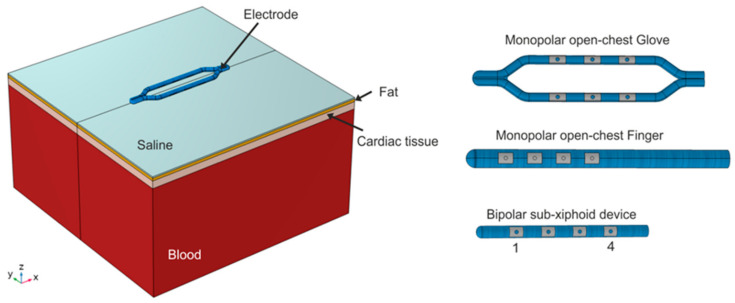
Geometry of the three-dimensional models, which includes different tissue layers below the devices (saline, fat, myocardial tissue, and blood). The layer geometry is the same for the three devices. On the right, the three devices modeled are as follows: monopolar open chest *Glove*, monopolar open-chest *Finger*, and bipolar subxiphoid device. The electrodes of the bipolar device are numbered for the ablation pairings.

Electric field models were created and solved numerically using the finite element method (FEM) with COMSOL Multiphysics (COMSOL, Burlington, MA, USA) for the monopolar open-chest *Glove* and *Finger* devices, as well as the new bipolar subxiphoid device. Figure 3 shows the three-dimensional model geometries built for these three devices. These models were based on different layers (saline, fat, cardiac tissue, and blood) that represent the elements below the device, placed in an epicardial approach. The GPs are embedded in epicardial fat pads; thus, the electric field strength within the fat is of particular interest. For all models, the thickness of the different layers were as follows: 0.5 mm for the saline, 1.0 mm for the fat, 2.7 mm for the cardiac tissue [21], and 40 mm for the blood. The devices were inserted 0.25 mm into the saline layer. The electrical conductivity of the tissues involved was modeled as a sigmoid function dependent on the electric field magnitude [22,23] to characterize the creation of pores during the PFA process. In this sense, the electrical conductivity increases during PFA as the cell becomes more permeable to electrical currents when PFA-induced pores are created. 

Simulations were performed by applying a pulse of 1000 V for 100 µs. For the *Finger* and open-chest *Glove*, the energy was applied in a monopolar mode, i.e., the energy was simulated with all the metal electrodes activated and the dispersive electrode placed on the bottom surface of the model. In the case of the bipolar device, the energy was simulated in a bipolar configuration between several different electrode pairings. For this device, the most distal electrode is defined as electrode 1 and the most proximal as electrode 4, as shown in Figure 3. For clarity, only bipolar pairings that provided characteristics similar to the monopolar devices are presented here. Specifically, BP1 is defined as electrodes 1 and 2 paired with 3 and 4, while BP2 is defined as electrodes 2 and 3 paired with 1 and 4.

The models provided data on electric field strength distribution; for purposes of design comparison, the linear dimensions and volume of tissue retained by selected field strength isolines were identified. Specifically, the isolines of 400 V/cm and 1000 V/cm were selected and corresponded approximately with a range of electroporation threshold values from published data [24,25]. Additionally, the models provided the current response passing through the target tissues. Energy input into the target GPs was calculated based on the voltage, cumulative pulse duration, and the current response:

Energy (J) = Voltage × Current × Time (1)

Taken together, the dimensions/volume of the tissue captured by the selected isolines, as well the computed current response, provide a basis for comparing the potential efficacy of the different device configurations.

### 2.3. Canine Study—Ablation Efficacy

Acute open-chest experiments were performed in 3 male mongrel canines (body weight of 25–35 kg) using the bipolar subxiphoid device. The protocol was approved in advance by the Mayo Clinic Institutional Animal Care and Use Committee in compliance with the Guide for Care and Use of Laboratory Animals. The animals were fasted overnight prior to the procedure. Anesthesia was induced with intramuscular tiletamine–zolazepam (5 mg/kg), followed by intravenous diazepam (0.5 mg/kg) and ketamine (10 mg/kg). The animals were intubated, supported with mechanical ventilation, and maintained on inhaled isoflurane (2–5%) with continuous intravenous fentanyl (2 mcg/kg/h) for additional analgesia. Adequate sedation and analgesia were ensured by monitoring for changes in heart rate and blood pressure exceeding 10% above baseline.

The skin was prepared by shaving the anterior chest and surface electrocardiogram (ECG) electrode sites. Using the percutaneous Seldinger technique, standard 9 Fr intravascular sheaths were placed in the right femoral and right jugular veins, and an 18 G arterial pressure line was placed in the left femoral artery.

A median sternotomy was performed using a combination of sharp and blunt dissections to expose the sternum which was then transected with trauma shears, and hemostasis was achieved with the use of electrocautery and bone wax applied to the cut bone edges. A sternal retractor was used to expose the mediastinum. The anterior visceral pericardium was incised, and using circumferentially anchored sutures, the heart was exposed. The pericardial recesses and thoracic cavity were kept free of excessive fluid accumulation by intermittent handheld suction.

A quadripolar standard curve, 8 Fr, 8 mm tip catheter (Blazer II XP, Boston Scientific Corp., Marlborough, MA, USA) was placed in the high right atrium (HRA) using femoral access and a 7 Fr deflectable duodecapolar catheter (Orbiter ST, Boston Scientific Corp., Marlborough, MA, USA) was inserted into the distal coronary sinus (CS) using jugular vein access. A 2-0 temporary cardiac pacing wire (Ethicon, Somerville, NJ, USA) was sutured into the base of the left atrial appendage (LAA), and a second wire was sutured into the subcutaneous tissue at the inferior margin of the sternotomy incision. Electrograms were recorded using the Prucka CardioLab EP system (General Electric Healthcare, Buckinghamshire, UK). Unipolar signals were recorded from catheter electrodes to the Wilson’s central terminal as reference. Bipolar pacing was performed from the distal electrodes of the HRA catheter and the most distal electrode pair that would allow atrial capture with the CS catheter—usually either pair 3–4 or 5–6. Monopolar pacing was performed from the LAA pacing wire (cathode) to the subcutaneous reference wire.

The atrial effective refractory period (AERP) was established at baseline in each of the 3 pacing sites (HRA, CS, and LAA) using an 8-cycle pacing train at a 500 ms cycle length with one extra stimulus. When heart rate remained consistently above 120 beats per minute, the pacing train cycle length was decreased to 450 ms. The pacing pulse width was 0.5 ms for all measurements. The pulse amplitude was 10 mA for the RA and LAA, while 4 mA was used for the CS. AERP measurements were repeated after all GP ablations completed, with a number of intermediate readings also recorded.

The AtriAN PEFG01 generator was used to deliver smooth waveform DC energy in pulses of 1000 V amplitude and 100 µs pulse width, in 10-pulse packets; pulses were monophasic and delivered in a bipolar manner between different electrode pairs on the bipolar catheter. Pulses were synchronized to the R wave of the surface ECG using a cardiac trigger monitor (Model 7600, Ivy Biomedical Systems, Brandford, CT, USA); one PFA pulse was delivered per heartbeat. A paralytic agent was not administered as it was expected that the bipolar configuration would produce less peripheral nerve stimulation than the previous monopolar version of the device.

The following six anatomical regions, known to contain GPs, were ablated; inferior left GP (ILGP), inferior right GP (IRGP), right superior GP (RSGP), transverse sinus GP (TSGP), left superior GP (LSGP) and ligament of Marshall GP (LMGP).

All studies were acute, and the animals were euthanized by induction of ventricular fibrillation through continuous DC stimulation at the end of each experimental procedure. Table 1 summarizes the combinations of pulse pairs and number of pulses delivered.

### 2.4. Histological Evaluation

Histology was performed on samples from the canine study only, with both ablation sites and collateral tissues/organs sampled. All slides were stained with hematoxylin and eosin (H–E) and Masson’s trichrome (MT) stains; slides from ablation sites were additionally stained with S-100 (for neuronal cell damage) and caspase 3 (for cellular apoptosis). All slides were assessed by an independent certified veterinary pathologist.

### 2.5. Porcine Study—Catheter Access and Navigation

**Figure 4:**
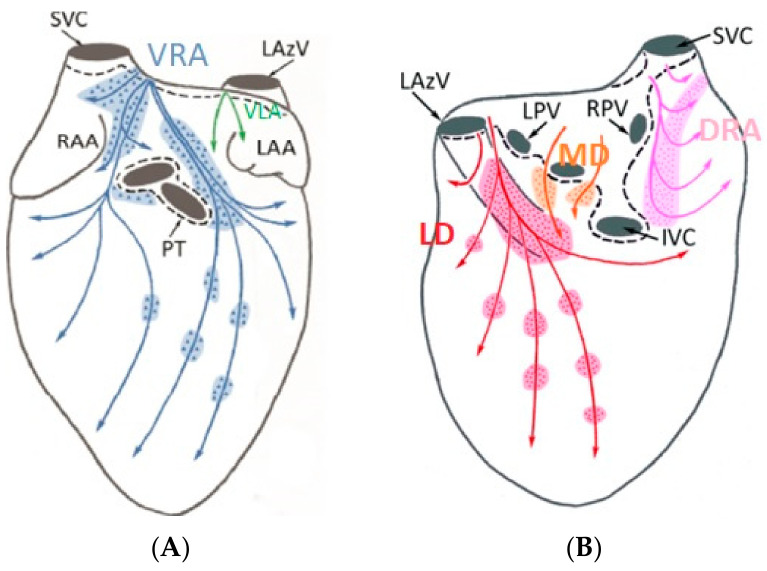
Anterior (**A**) and posterior (**B**) GPs in the porcine model. (Adapted from Aksu et al., J Cardiovasc Electrophysiol. 2021 [26]).

This study was performed on two healthy porcine subjects (40–50 kg) primarily to assess pericardial access and navigation techniques for the subxiphoid bipolar prototype device. Recognizing that the GPs locations and electrophysiology responses in the porcine model are different to canines or humans, AERP extensions were not assessed in this part of the study, though bipolar ablations were performed. The study was reviewed and approved by an external ethical committee for compliance with regulations prior to study initiation. The protocol was approved and carried out by the Center for Cardiovascular Research and Development, American Heart of Poland. After overnight fasting, the animals were anesthetized using a combination of ketamine (10–20 mg/kg, IM), xylazine (0.05–0.2 mg/kg, IM), and atropine (0.02–0.05 mg/kg, SC). After intubation, anesthesia was maintained using 1–3% isoflurane with 100% oxygen, inhaled, and propofol 0.1–0.2 mg/kg/min, intravenously. A bolus of fentanyl was administered to increase the depth of general anesthesia and provide analgesia. Subxiphoid epicardial anterior access into the pericardial space was obtained under fluoroscopic guidance using conventional Touhy needles and introducer sheaths. A guidewire was initially passed through to confirm proper entry to the pericardial space, by visualizing the guidewire tracking around the heart epicardial surface under fluoroscopy. A 0.032” guidewire was then used to aid positioning of an 8.5 Fr Agilis EPI steerable sheath (Abbott Cardiovascular, Santa Clara, CA, USA). The prototype ablation catheter was positioned within the steerable sheath, providing additional navigation capability, in conjunction with the deflectable tip of the catheter. Both the dorsal right atrial (DRA) and ventral right atrial (VRA) GPs were accessed under fluoroscopic guidance, with visualization enhanced by contrast injection into the pericardial space. The location of these GPs in the porcine model is schematically shown in Figure 4. Identical to the canine ablations, the AtriAN PEFG01 generator was then used to deliver smooth waveform DC energy in pulses of 1000 V amplitude and 100 µs pulse width, in 10-pulse packets; pulses were monophasic and delivered using the bipolar BP2 configuration, with 120 repeat pulses per GP. Given the relatively large area covered by the VRA GP, the catheter was repositioned and a second set of 120 pulses was delivered at this GP location only. The animals were sacrificed immediately after the experiments.

## 3. Results

### 3.1. Electric Field Models

**Figure 5:**
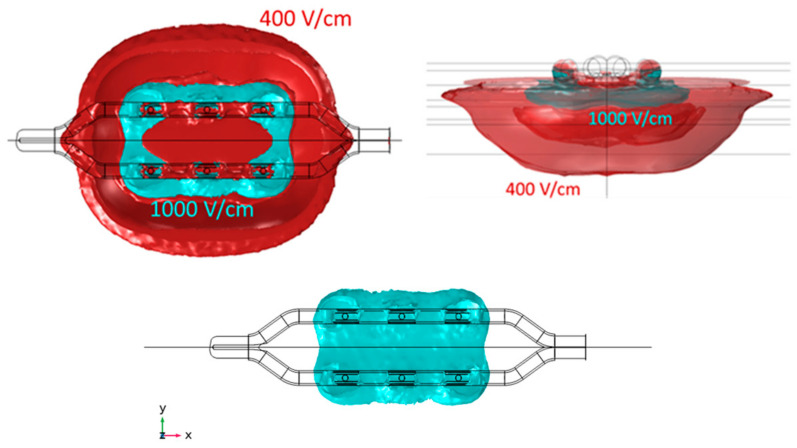
Geometry of the electric field within 400 V/cm and 1000 V/cm isolines for the monopolar open-chest *Glove* device. The 1000 V/cm volume is shown separately, as the merged profiles obscure the true shape of this. Length is obtained in the X direction, width in Y direction, and depth in Z direction.

**Figure 6:**
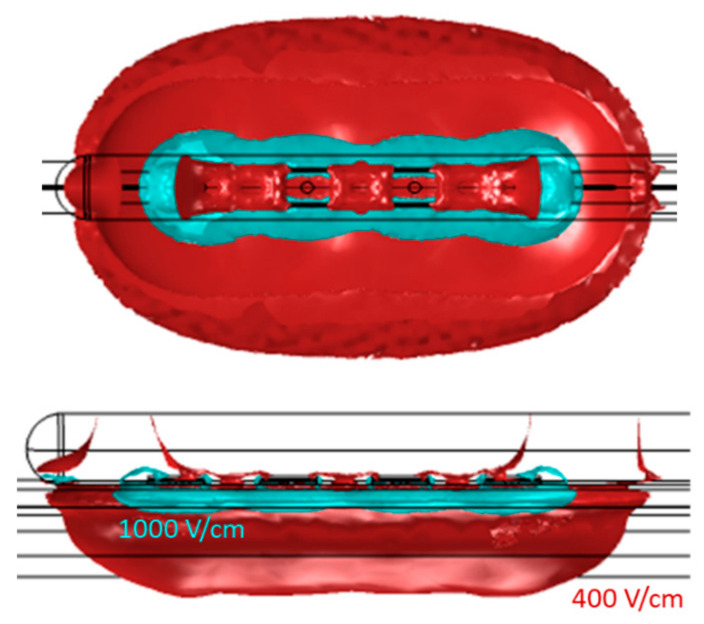
Geometry of the electric field within 400 V/cm and 1000 V/cm isolines for the monopolar open-chest *Finger* device.

**Figure 7:**
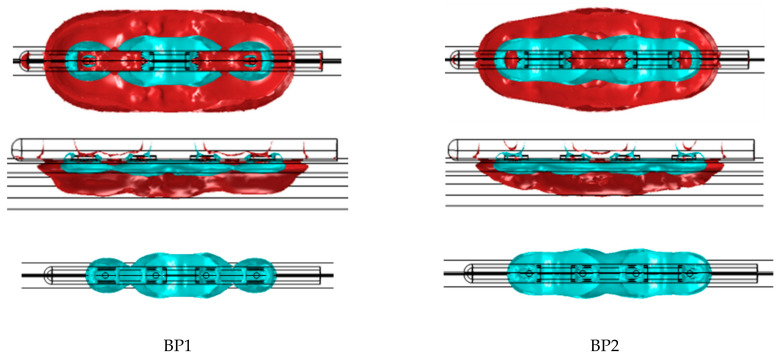
Geometry of the electric field within 400 V/cm and 1000 V/cm isolines for the bipolar B1 and B2 configurations of the subxiphoid device. The 1000 V/cm volume is shown separately as the merged profiles obscure the true shape of this.

Table 2 presents the dimensions and volume captured within the isolines of 400 V/cm and 1000 V/cm, as well as the shares of this volume residing within both the epicardial fat and cardiac tissue (note that the total volume also captures the layer of saline over the fat and blood beyond the cardiac tissue; these volumes are not considered in this assessment). The total currents computed from the model are also included. Figure 5 and Figure 6 illustrate the isoline volumes for the two monopolar (open-chest) devices, while Figure 7 shows this for the BP1 and BP2 bipolar (subxiphoid) configurations. The dimensions and shape of the electric fields computed for the bipolar modes compare well to the monopolar *Finger*, particularly at the 1000 V/cm isoline. Considering that epicardial fat is the target tissue, then bipolar mode BP2 is the closest to capturing a volume comparable to the monopolar configuration.

**Figure 8:**
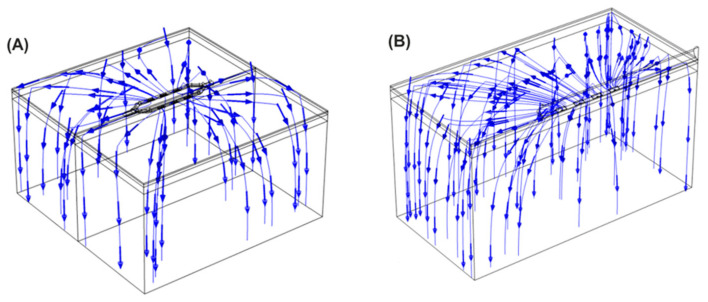
Current streamlines for the monopolar *Glove* device (**A**) and *Finger* device (**B**).

**Figure 9:**
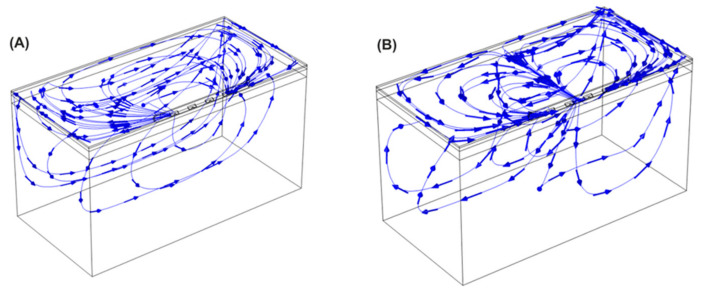
Current streamlines for the bipolar device applying the energy in BP1 (**A**) and BP2 (**B**) configurations.

Figure 8 shows the current streamlines for the monopolar open-chest *Glove* and the monopolar open-chest *Finger*. For the bipolar configurations, the different current paths are shown in Figure 9. The current data show that a higher number of the BP pulses would be needed to achieve the same overall energy input as 60 of the monopolar pulses. For example, 60 of the *Glove* monopolar pulses deliver 21.2 joules; it would take at least 80 BP2 pulses to achieve approximately the same energy input (22.6 joules). Alternatively, a mixed configuration of 50 BP1 plus 40 BP2 would provide 21.4 joules, also comparable to the monopolar energy (pulse trains are delivered in a fixed sequence of 10 pulses, hence the use of decades for pulse selection).

### 3.2. Canine Study—Ablation Efficacy

A sternotomy was successfully performed, and pulsed electric fields were successfully delivered to all the target sites in each animal. Peripheral muscle stimulation was not quantitatively assessed but was barely perceptible, though no paralytic agent was administered. Table 3 presents the average per pulse current response (recorded from the generator) and energy input per GP site for the BP1 and BP2 pulses delivered in each animal. Overall averages are also presented.

While the overall pulse averages may mask some differences between GPs, these values are used for computing the total energy imparted per GP and per animal in the ablation sequences, as shown in Table 4. Table 5 presents the baseline and postablation AERP values for each animal, at the three measurement locations. Average AERPs are used to calculate the AERP extension (%) between the baseline and postablation. With the exception of the LAA in animal 1, all locations showed an AERP extension; the average AERP extension ranged from 22% to 96%. While the lowest energy input (194.6 J) is associated with the shortest AERP extension (22%), there is no evident correlation overall between energy and AERP extension due to the small number of data points.

### 3.3. Histologic Evaluation

**Figure 10:**
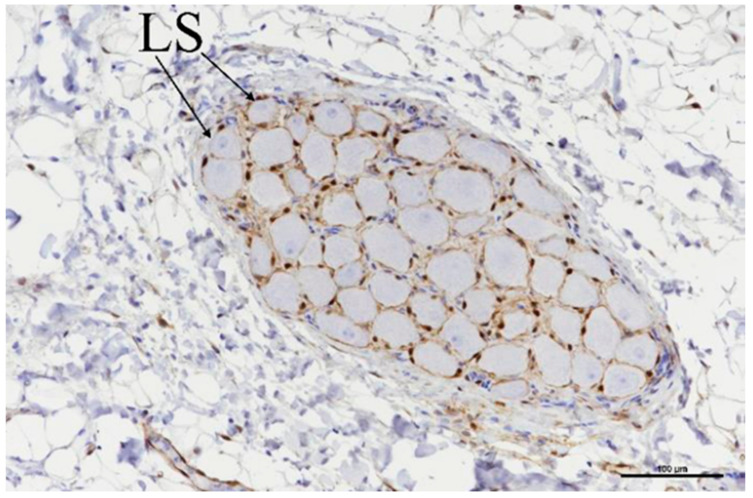
Ganglia showing neuronal cell bodies with loss of S-100 staining (LS). Scale bar = 100 µm.

**Figure 11:**
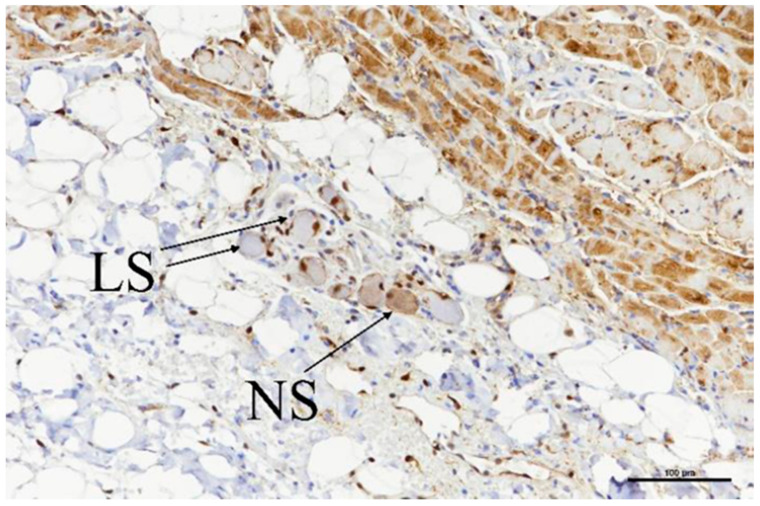
Ganglia showing neuronal cell bodies with loss of S-100 staining (LS) as well as some with normal staining (NS) retained. Scale bar = 100 µm.

Ganglia were located in a total of nine slides (Table 6), and all nine showed evidence of acute ablation damage on the S100 staining; on a damage scale of 0–4, eight had a score of 4 and one had a score of 3. Figure 10 shows an example of a ganglion with complete loss of S100 staining within the neuron cell bodies. Figure 11 shows the sample with a score of 3; this serves as a useful comparison with both ablated and nonablated cell bodies visible, i.e., the nonablated structures showing the S100 uptake (light brown). 

While the loss of neuronal cell functionality was confirmed, all staining techniques showed the neuronal cells to be structurally intact. There was no evidence of caspase 3 stain uptake in most of the ablated ganglia, suggesting that apoptosis was not the predominant mechanism of cell death. One exception was an ILGP slice that had been ablated with 60 of the BP2 pulses, which had a caspase 3 stain uptake with a damage score of 2.

**Figure 12:**
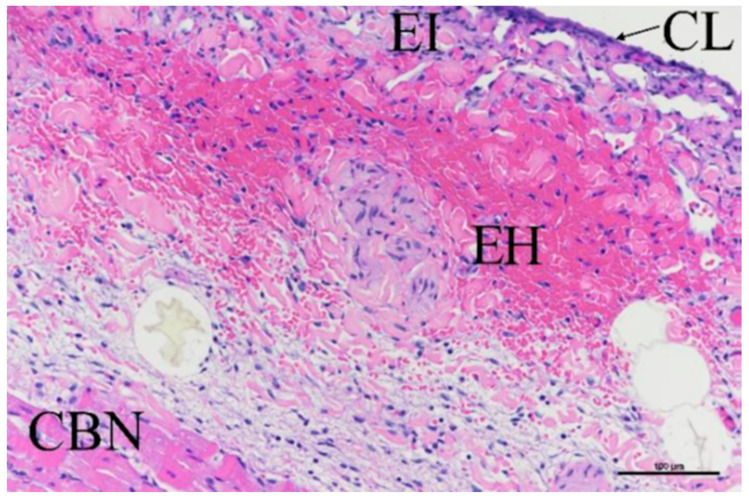
H–E stain showing myocardium damage at ablation site, showing contraction band necrosis, epicardial hemorrhage (EH), inflammation (EI), and collagenolysis (CL). Scale bar = 100 µm.

A total of 27 slides evaluated the myocardial damage at the ablation sites; for all characteristics assessed, the majority of the damage scores were 0 or 1 on the 0–4 scale. There were three scores of 3 identified for cardiomyocyte contraction band necrosis (CBN); these were all associated with the 50 BP1 + 40 BP2 treatment, though it is more likely that this was associated with catheter handling rather than the ablation parameters per se. Figure 12 shows a worst case of CBN as well as traces of epicardial hemorrhage, inflammation, and collagenolysis. 

In relation to adjacent tissues and organs, most scores were 0, 1, or 2 using the same damage scale. There was a score of 3 recorded on LAA samples from the 50 BP1 + 40 BP2 treatment; of note, the LAA was used as the site of pacing wire attachment for AERP measurements. It is possible that the additional handling and attachment caused tissue damage beyond the evident attachment point. Out of four esophageal tissues assessed, one had a damage score of 2 for acute adventitial hemorrhage, adjacent to an ILGP ablation. It is unlikely to be due to “over-ablation”, as in this instance, the ILGP was treated with only 60 pulses and had a lower energy input per GP compared to other ILGP and IRGP ablations: 21.78 J versus 32.44 J, respectively. Four left phrenic nerve samples were assessed, and no damage was observed, all scoring 0 on the 0–4 scale.

### 3.4. Porcine Study—Catheter Access and Navigation

**Figure 13:**
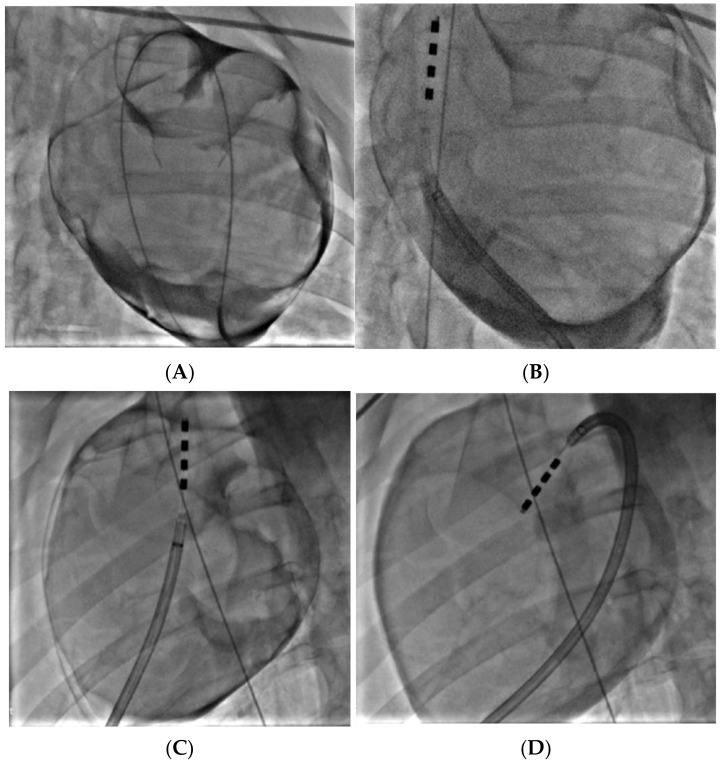
Confirmation of proper epicardial access, with the guidewire visible in the pericardial space around the entire silhouette of the heart (**A**). Prototype catheter positioned at the VRA GP (**B**). Catheter positioned at the DRA GP (**C**) and VRA GP (**D**); guidewire in vena cava to confirm anatomical position.

Subxiphoid anterior access was achieved in both animals and the prototype catheter was successfully navigated to the target treatment sites at the DRA and VRA GPs. Figure 13A shows confirmation of access into the pericardial space before navigation of the device to the target sites, while Figure 13B–D shows the ablation device in position at the treatment sites, having been deployed from the distal tip of the steerable sheath. Overall, the catheter worked well, and the four electrodes were clearly visible under fluoroscopy, which aided navigation. While the tip of the catheter was not highly flexible, the additional steerability provided by the access sheath ensured that all sites could be reached. The correct orientation of the electrodes and adequate tissue contact were confirmed though fluoroscopy and local electrogram readings. 

**Figure 14:**
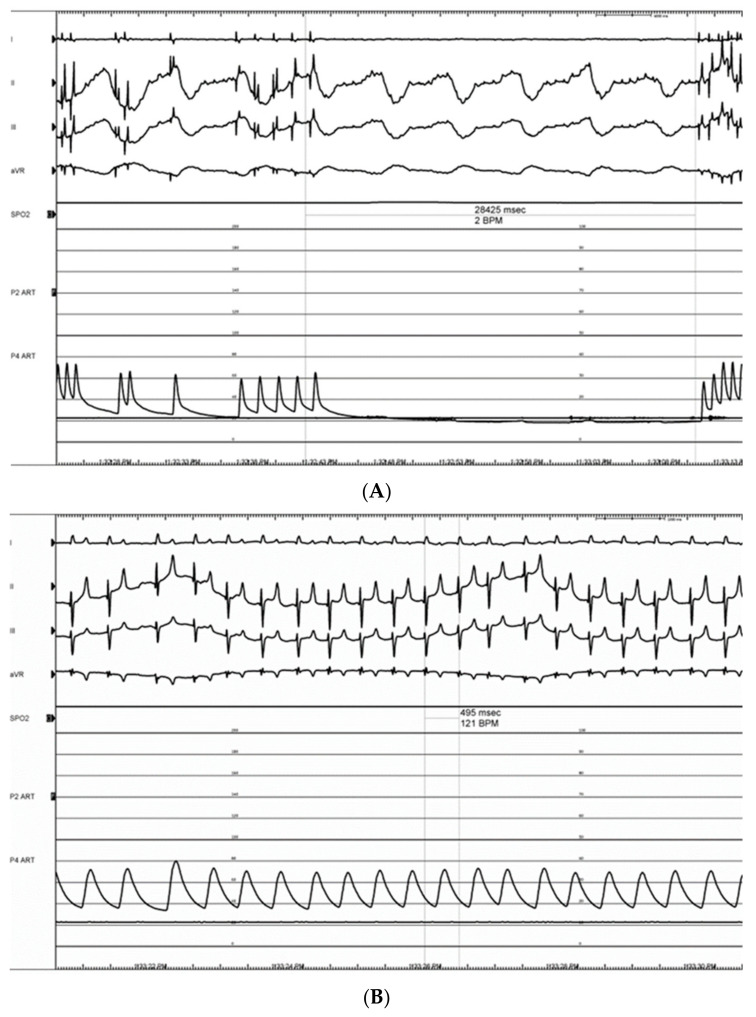
Electrograms showing long (28.4 s) transient complete AV-block (III grades) episode during energy delivery at the VRA GP (refer to Figure 13D) (**A**). This was followed by full recovery of atrioventricular conduction, followed by sinus tachycardia, presumably triggered by activating the sympathetic component of the autonomic nervous system (**B**).

**Figure 15:**
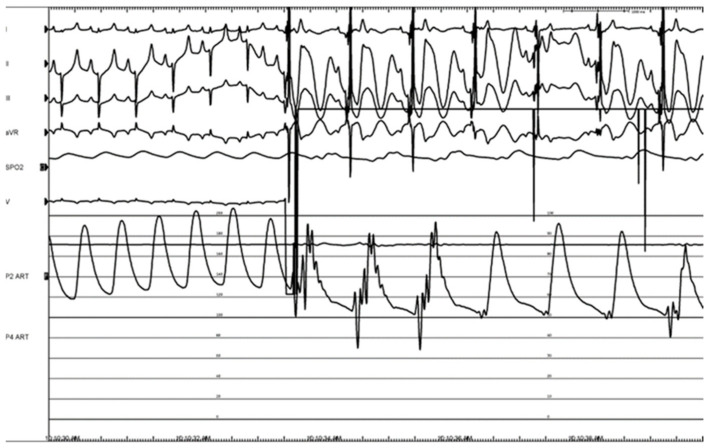
Bradycardia with a noticeable drop in blood pressure during therapy, during energy delivery at the VRA GP.

Pulsed electric field ablation energy was successfully delivered to all target sites as planned using the bipolar BP2 configuration. Significant vagal responses were noted during the delivery of the ablation pulses; this included cases of transient complete AV-block (Figure 14A,B) as well as bradycardia (Figure 15). These were primarily associated with VRA GP ablations rather than DRA GP ablations, but all recovered fully and spontaneously immediately after the energy delivery.

Stimulation at the GPs, before and after ablation (using a Biotronik Reocor S external pacemaker at 1000 BPM, 15–17 V), caused a temporary decrease in the heart rate of about 5 BPM. This decrease was absent when restimulated after ablation.

## 4. Discussion

The involvement of GPs in both the initiation and propagation of AF has long been appreciated [27], though the precise contribution to AF recurrence after ablation has not been clearly elucidated, with randomized trials giving mixed results. A promising early trial explored the addition of endocardial GP ablation to conventional endocardial PVI; at two-year follow-ups, freedom from AF was 48% for GP ablation alone, 56% from PVI alone, and 74% for PVI combined with GP ablation [28]. It was, however, noted that GP ablation increased the risk of left atrial tachycardia and flutter, probably due to excessive ablation of the myocardium in the vicinity of the GPs, creating the substrate for macro-reentry arrhythmias. This observation of increased rates of atrial tachycardia was also reported in a thoracoscopic GP ablation study that compared PVI to PVI with GP ablation: all ablations were epicardial and thoracoscopic [29]. The technique of the current report enables the selective epicardial ablation of GPs without notably damaging the surrounding myocardium [30]; in the context of AF, it therefore has the potential to better identify the contribution that GPs and their ablation provides. Importantly, one could hypothesize that eliminating the GPs with roles in AF initiation and potentiation could provide for better long-term outcomes when combined with PFA PVI. This is particularly relevant in the context of a ceiling effect in outcomes from PVI trials, and the evidence suggesting that these new PFA PVI devices are not ablating the GPs, and therefore, losing the contribution that GP ablation may provide in the overall outcome for the patient.

The device currently in development is an updated iteration of one already successfully used in open-chest preclinical and clinical studies; catheter handle and shaft refinements provide for minimally invasive access and navigation, while a change from monopolar to bipolar energy delivery is aimed at improving the efficiency of energy delivery and reducing peripheral nerve stimulation by the pulsed electric field.

The results from the electric field modeling helped to identify bipolar parameters that can provide the same energy input to the GPs as already used clinically in the monopolar configuration. At an initial level, the dimensions and shape of the electric field within the selected isolines provide a good comparison (Table 2 and Figure 5, Figure 6 and Figure 7) between the monopolar and bipolar configurations. The 1000 V/cm isoline is probably the most clinically relevant in the context of obtaining irreversible electroporation. Perhaps not surprisingly, the width of the field for the bipolar configurations is approximately half that of the monopolar *Glove*; thus, from the perspective of ablating within the oblique sinus, the new bipolar *Finger*, used individually at the IRGP and ILGP, may possibly replace the monopolar *Glove* device. This consolidation into a single device is important from an engineering and cost perspective, as well as simplifying the whole approach for the clinician. The depth of ablation with bipolar configurations is approximately 0.6 mm deeper than monopolar, though importantly, this does not necessarily mean that more cardiac tissue is captured; the model data suggest that the volume of cardiac tissue is much lower with bipolar configurations, possibly enhancing selectivity effects, i.e., reducing the potential for myocardial ablation. Additionally, the length of the 1000 V/cm field is notably more for the bipolar configurations, again suggesting that this device could be at least as effective as the monopolar *Glove* when used individually for the IRGP and ILGP. In comparing the two bipolar configurations, while they are similar, BP2 gives higher volumes of tissue capture, possibly giving higher efficacy. 

The computational data provided guidance for the initial selection of pulse sequences used in the canine study as indicated in Table 4. Of note, there is a difference between the computed current values (Table 2) and those recorded experimentally (Table 3); considering the assumptions and limitations of the model (including the fixed dimensions for tissue thicknesses), this difference is indeed very small, and such differences and variations are to be expected in experimental data. 

The AERP extensions recorded in all three canines compare favorably to previous monopolar data, which were typically around 20% extensions in both preclinical [19] and clinical studies [20]. The higher extensions for the bipolar configurations may be due to a more focused delivery of the energy into the fat and GPs, with less being absorbed elsewhere across the anatomy as may happen with monopolar configurations, when the energy passes between the catheter and a dispersive pad on the subject’s lumbar region. The current streamlines shown in Figure 8 and Figure 9 would support this theory. While a change in tissue refractoriness is a useful surrogate for the verification of GP ablation, the high variability in the extension values observed here indicates that it may not be the optimum methodology in the long term. It is possible that the technique of extra-cardiac vagal stimulation (ECVS) may provide a more reliable measure of baseline and postablation vagal tone [31]. This technique requires catheter access into both left and right internal jugular veins in order to achieve proximity to the vagus nerve for stimulation; additionally, the optimization of stimulation parameters for each patient may be required. However, the technique has been successfully used in several studies of GP ablation for the treatment of both atrial fibrillation and syncope [32].

The histology evaluations generally support the observed acute AERP extension in terms of loss of neuronal cell functionality within the GPs evaluated. The observed structural integrity of the neuronal cells may be related to the acute sacrifice timepoint; a delayed sacrifice may have seen a temporal effect of the electroporation. Damage to the myocardium and collateral tissues was observed at a low level; while this will continue to be assessed in more detail going forward, in most instances, it appeared to be related to device handling rather than the ablation parameters. 

The porcine study on access and navigation worked very well using the prototype. Safely achieving subxiphoid access with standard Touhy needles was important to observe, though it is worth noting that there are also a number of devices and methods in development that should make this technique even easier to perform safely [33,34,35]. The catheter was also demonstrated to be fully compatible with the commercially available Agilis EPI steerable sheath (Abbott Cardiovascular, Santa Clara, CA, USA), giving optimum navigation and positioning. The vagal response observed during energy delivery in the porcine model merits further consideration; this has not been observed during GP ablations in the open-chest first-in-human studies or during any previous canine study—open-chest or percutaneous. It has, however, been widely reported during endocardial pulsed field PVI in clinical settings [36,37], where it is considered to be on-going capture of the GPs by the electric field at a level sufficient to stimulate but not sufficient to cause ablation. The absence of a vagal response during epicardial GP ablation in both humans and canines is at least consistent in that the GP locations and conduction systems in both are considered comparable, but different to the porcine model [26,38]. Additionally, in these porcine ablations, it was noted that the vagal response was primarily associated with the VRA GP rather than the DRA GP. Perhaps the relatively large and diffusely spread area of the VRA GP meant that regions of it were too far from the ablation electrodes and were continuously stimulated, rather than ablated. Directly related to this, it is recognized that the porcine AV node is more heavily innervated than that of humans or canines [39]; it is possible that this also contributes to the ease of stimulation and the significant vagal responses observed. In any event, going forward with this development, while both canine and porcine studies will be performed, the data from the canine models will be considered more representative of the human anatomy and electrophysiology.

## 5. Limitations

There are a number of limitations to our studies, most notably the use of animal models, where extrapolation to human anatomy and physiology cannot be assumed. The electric field models used a fat thickness of only 1 mm, and while the initial canines and humans studied had minimal epicardial fat, further modeling will be needed to assess the energy requirements for thicker layers of epicardial fat.

This was an acute study only, with a small number of animals. Further studies with increased sample sizes and chronic timepoints will be needed to better assess the durability of the treatment. The ablation parameters were initially assessed in an open-chest configuration for comparison to previous open-chest work—all future studies will be performed with subxiphoid access.

The device used was a first prototype with more engineering development expected; the bipolar parameters will also likely need further optimization.

The canines assessed for tissue refractoriness were all healthy before the treatment. An AF animal model will be considered for further studies as well as assessments of AF inducibility, both acutely and chronically.

## 6. Conclusions

Epicardial GP ablation using subxiphoid percutaneous access, with a bipolar pulsed field ablation device, has been demonstrated to be feasible and effective in these preliminary computational and preclinical studies. Ablation parameters have been identified that successfully ablate the GP neuronal cell bodies, with minimal damage to the myocardium or other collateral structures. The device can be used to navigate to target GP sites, and postablation extensions in the atrial tissue refractoriness point to the potential for treating atrial fibrillation. 

## Figures and Tables

**Table 1:** Bipolar electrode pairs and number of pulses delivered to each GP site. (BP1 is defined as electrodes 1 and 2 paired with 3 and 4, while BP2 is defined as electrodes 2 and 3 paired with 1 and 4).

Animal	Target GPs	Bipolar Pairs/Sequence
**1**	ILGP, IRGP, RSGP, TSGP, LSGP, LMGP	50 BP1 pulses + 40 BP2 pulses
**2**	ILGP, IRGP, RSGP, TSGP, LSGP, LMGP	120 BP2 pulses
**3**	ILGP, IRGP	60 BP2 pulses
	RSGP, TSGP, LSGP, LMGP	120 BP2 pulses

**Table 2:** Dimensions and volumes within the 400 V/cm and 1000 V/cm isolines for all designs and configurations.

	Isoline (V/cm)	Length (mm)	Width (mm)	Depth (mm)	Total Volume (mm^3^)	Fat Volume (mm^3^)	Myocardium Volume (mm^3^)	Total Current (A)
**Monopolar Finger**	400	33.84	19.80	6.38	2235.80	551.72	1020.90	3.47
	1000	25.56	6.71	1.81	161.35	141.14	0.39	
**Monopolar Glove**	400	30.44	24.16	6.93	2172.70	559.29	848.61	3.54
	1000	17.95	11.77	1.32	219.47	197.17	6.41	
**Bipolar BP1**	400	29.08	11.77	4.09	868.88	297.48	381.84	2.03
	1000	24.21	5.72	1.91	144.46	94.71	1.84	
**Bipolar BP2**	400	28.67	12.31	4.05	850.34	269.50	360.36	2.82
	1000	24.45	5.92	1.91	202.08	111.96	2.58	

**Table 3:** Current and energy responses per pulse for each GP site using the bipolar configurations.

		ILGP	IRGP	RSGP	TSGP	LSGP	LMGP	Overall Average
**Animal 1**	**BP1 Current** (A)	4.83	2.55	3.84	3.09	3.42	2.44	3.36
	**BP1 Energy** (J)	0.483	0.255	0.384	0.309	0.342	0.244	0.336
	**BP2 Current** (A)	6.05	2.68	4.59	3.77	4.06	2.28	3.91
	**BP2 Energy** (J)	0.605	0.268	0.459	0.377	0.406	0.228	0.391
**Animal 2**	**BP2 Current** (A)	5.35	5.87	5.80	4.11	4.39	5.55	5.18
	**BP2 Energy** (J)	0.535	0.587	0.580	0.411	0.439	0.555	0.518
**Animal 3**	**BP2 Current** (A)	3.93	3.72	2.21	4.31	3.86	3.76	3.63
	**BP2 Energy** (J)	0.393	0.372	0.221	0.431	0.386	0.376	0.363

**Table 4:** Energy delivered per GP site and to all (six) GP sites.

	Pulse Description	Overall Energy per GP (J)	Overall Energy per Animal (J)
**Animal 1**	50 BP1 + 40 BP2	32.44	194.6
**Animal 2**	120 BP2	62.16	373.0
**Animal 3**	60 BP2 for ILGP and IRGP	21.78	217.8
	120 B2 for all other GPs	43.56	

**Table 5:** AERP values at baseline and postablation for each animal at the three locations.

		RA (ms)	CS (ms)	LAA (ms)	Average (ms)	AERP Extension (%)
**Animal 1**	Baseline	90	100	80	**90**	**22**
	Postablation	120	140	70	**110**	
**Animal 2**	Baseline	110	-	110	**110**	**68**
	Postablation	190	-	180	**185**	
**Animal 3**	Baseline	80	70	90	**80**	**96**
	Postablation	140	140	190	**157**	

**Table 6:** Slides with ganglia detected, indicating GP location and ablation treatment delivered.

Target GP	No. of Slides with Evaluated GPs	Treatment
**ILGP**	2	60 BP2, 50 BP1 + 40 BP2
**IRGP**	2	Both 50 BP1 + 40 BP2
**TSGP**	2	120 BP2, 50 BP1 + 40 BP2
**LSGP**	1	50 BP1 + 40 BP2
**LMGP**	2	120 BP2, 50 BP1 + 40 BP2

## Data Availability

The data underlying this article will be shared upon reasonable request to the corresponding author.
